# Extracellular matrix remodeling modifies structural responses to ventilator-induced lung injury: a multiscale correlative imaging study

**DOI:** 10.1186/s12931-026-03807-y

**Published:** 2026-07-13

**Authors:** Md Motiur Rahman Sagar, Lorenzo D’Amico, Richard T. Deyhle, Ruth Meyer, Luca Fardin, Irma Mahmutovic Persson, Jose Luis Cercos-Pita, Gaetano Perchiazzi, Sarah Köster, Claudia V. Benke, Frauke Alves, Giuliana Tromba, Lars E. Olsson, Sam Bayat, Christian Dullin

**Affiliations:** 1 Translational Molecular Imaging, Max-Planck-Institute for Multidisciplinary Sciences, Goettingen, Germany; 2 Elettra - Sincrotrone Trieste S.C.p.A., Trieste, Italy; 3 Department of Translational Medicine, Lund University, Medical Radiation Physics, Malmö, Sweden; 4 Institute for X-Ray Physics, University of Göttingen, Göttingen, Germany; 5 Hedenstierna Laboratory, Department of Surgical Sciences, Uppsala University, Uppsala, Sweden; 6 Department of Diagnostic and Interventional Radiology, Heidelberg University Hospital, Heidelberg, Germany; 7 Translational Lung Research Center Heidelberg (TLRC), German Center for Lung Research (DZL), Heidelberg, Germany; 8 Department for Clinical and Interventional Radiology, University Medical Center Goettingen, Goettingen, Germany; 9 Clinic for Haematology and Medical Oncology, University Medical Center Goettingen, Goettingen, Germany; 10 Inserm UA07 STROBE Laboratory, Université Grenoble Alpes, Grenoble, France

**Keywords:** Propagation-based imaging, Ventilator-induced lung injury, Correlative imaging, FFPE lung tissue, Lung fibrosis

## Abstract

**Background:**

Mechanical ventilation (MV) can induce or exacerbate ventilator-induced lung injury (VILI), particularly in mechanically heterogeneous lungs with pre-existing injury.

**Methods:**

We investigated VILI in a rat model of bleomycin-induced lung injury and compared it with healthy controls using a combined in-vivo and ex-vivo imaging approach. Previously acquired in-vivo data from four-dimensional (4D) phase-contrast synchrotron micro-computed tomography (micro-CT) and forced oscillation measurements showed increased lung elastance and reduced local acinar deformation in bleomycin-induced injured lungs at baseline and after injurious MV. To identify structural and mechanical correlates, we performed automated three-dimensional (3D) pore analysis and atomic force microscopy (AFM) on formalin-fixed, paraffin-embedded lung tissue, complemented by histology and spatial co-registration.

**Results:**

Ex-vivo analysis revealed pronounced airspace enlargement after both injurious MV of healthy lungs, and in bleomycin-injured lungs with inflammation and early fibrotic changes, with the strongest cumulative effect in combined bleomycin and VILI. AFM demonstrated region-specific mechanical responses, and correlation analyses linked pore geometry and nanoscale stiffness to in-vivo lung mechanics. Spatial analysis further showed co-localization of VILI-associated airspace damage with injured regions.

**Conclusions:**

Extracellular matrix remodelling modifies the lung’s response to injurious mechanical ventilation, with VILI-associated airspace damage preferentially co-localising with regions of pre-existing matrix injury. This multiscale correlative approach provides mechanistic insight into the interplay between lung injury and VILI and informs ventilation strategies in structurally altered lungs.

## Introduction

Mechanical ventilation (MV) plays a critical role in patient care by supporting gas exchange and alleviating the work of breathing in acute respiratory failure. Unlike natural breathing, which generates negative pressure in the chest upon inspiration, MV generally employs positive pressure. However, positive pressure ventilation can induce excessive stress and strain in the lung parenchyma, causing or worsening pre-existing lung injury, a phenomenon known as ventilator-induced lung injury (VILI) [1]. Despite decades of research and the development of lung-protective ventilation strategies, VILI remains a major clinical challenge with significant morbidity and mortality [2–4].

A central limitation in understanding and preventing VILI is the incomplete knowledge of how mechanical forces are distributed and propagated within heterogeneous lung tissue at the microscale. While global parameters such as tidal volume and plateau pressure are well established, they fail to capture local variations in tissue mechanics and structure that are thought to drive regional overdistension and injury [5]. In particular, the relationship between local tissue morphology, viscoelastic properties, and mechanical stress distribution remains poorly understood, especially in the presence of pre-existing lung injury.

To address this gap, experimental models that reproduce key features of lung injury are essential. In this study, we investigate the effect of injurious MV on microscopic tissue morphology and stiffness in healthy control rat lungs and in lungs 7 days after bleomycin-induced injury. Our approach is motivated by the need to link regional lung mechanics with underlying structural and material properties across scales. To this end, we combine imaging and biomechanical assessment techniques that are sensitive to complementary aspects of lung tissue behavior. High-resolution imaging enables the characterization of regional deformation and structural heterogeneity, while nanoscale mechanical probing provides direct measurements of local tissue stiffness. By integrating these perspectives, we aim to better understand how pre-existing injury alters the mechanical response of lung tissue to ventilation and contributes to the development of VILI.

The bleomycin-induced lung injury model is widely used due to its reproducible progression from acute inflammation to fibrotic remodeling. Importantly, the temporal evolution of this model must be considered when interpreting mechanical alterations. In this study, we focus on a 7-day time point after bleomycin administration, which represents a transitional phase characterized by pronounced inflammation, epithelial damage, and the onset of extracellular matrix deposition, preceding the development of fully established fibrosis [6]. This early stage provides a relevant framework to investigate how early structural remodeling influences susceptibility to VILI.

A more detailed understanding of the interplay between lung structure and mechanics at the microscale can help explain why injured lungs respond heterogeneously to ventilation and could inform the development of more effective and individualized ventilation strategies.

## Methods

### In-vivo respiratory mechanics and strain mapping

Previously published in-vivo datasets from the same cohort were included to enable correlation with the newly acquired ex-vivo measurements [7]. Respiratory mechanics in the cited work was assessed by the forced oscillation technique (FOT) using an ad hoc setup.

Dynamic four-dimensional synchrotron phase-contrast micro-CT was used to generate quantitative maps of local lung strain during tidal ventilation. These datasets provided complementary organ-scale and acinar-scale measures of lung mechanical behavior for correlation with the ex-vivo structural and AFM measurements. Direct in-vivo–ex-vivo correlations were restricted to the Bleo-VILI cohort. Additional methodological details on the acquisition and analysis of respiratory mechanics and imaging data are provided in the Supplementary Methods.

In Deyhle et al. [7] control and bleomycin-injured rats, 7 days post-intratracheal instillation, FOT was performed at baseline, followed by 4DCT image acquisition for 9 min. After baseline data acquisition, injurious ventilation was initiated by increasing peak respiratory pressure to 41 ± 2 cmH2O, with a PEEP of 0 while respiratory rate was reduced to 30 bpm for 20 min. The measurements including lung mechanics and image acquisition were then repeated post-injurious ventilation. At the end of the measurements, the animals were euthanized by intraperitoneal injection of pentobarbital sodium (Dolethal, 200 mg/kg, Vetoquinol, Lure, France) and the heart and lungs were dissected and removed en bloc for histological analysis.

### Overview of the multiscale correlative workflow

To investigate how extracellular matrix remodeling modifies susceptibility to ventilator-induced lung injury (VILI), we implemented a correlative multiscale imaging workflow integrating in-vivo respiratory mechanics and strain mapping with ex-vivo structural and biomechanical analysis. Synchrotron phase-contrast micro-CT was used to quantify three-dimensional airspace architecture in formalin-fixed paraffin-embedded (FFPE) lung tissue, while guided sectioning enabled spatially registered histology and atomic force microscopy (AFM) measurements from corresponding regions. FFPE tissue enabled stable multiscale imaging, guided sectioning, and spatial co-registration across modalities while preserving tissue architecture. Automated pore analysis provided quantitative descriptors of local airspace remodeling, AFM quantified microscale tissue stiffness, and histology enabled assessment of consolidation and tissue injury. Together, these complementary techniques enabled integration of organ-scale lung mechanics with microscale structural remodeling and local biomechanical alterations.

### Experimental setup and sample preparation

The experiments were performed on 20 Sprague-Dawley rats (average weight: 399±26 g), which were divided into four experimental groups: healthy controls after protective mechanical ventilation (Con), healthy controls after injurious ventilation (Con-VILI), bleomycin-treated lungs after protective ventilation (Bleo), and bleomycin-treated lungs after injurious ventilation (Bleo-VILI). Bleomycin-induced injury was established by intratracheal instillation of bleomycin seven days prior to imaging.

A subset of animals underwent in-vivo synchrotron phase-contrast micro-CT imaging and respiratory mechanics measurements before and after induction of VILI using high-pressure mechanical ventilation, as previously described by Deyhle et al. [7]. Following the experiments, lungs were fixed at constant airway pressure, dehydrated, and embedded in paraffin. The left lung lobe was divided into upper and lower regions for subsequent ex-vivo analysis. Additional experimental details are provided in the Supplementary Methods.

### Phase-contrast micro-CT

FFPE lung specimens were imaged at the SYRMEP beamline [8] using propagation-based synchrotron phase-contrast micro-CT. Reconstructed datasets with isotropic 2 µm voxel size were used for three-dimensional structural analysis, guided sectioning, and spatial registration with histology and AFM. Detailed acquisition and reconstruction parameters are provided in the Supplementary Methods.

### Quantification of airspace remodeling

Automated pore analysis was performed on reconstructed phase-contrast micro-CT datasets using PoreSpy [9]. Segmented airspaces were separated into isolated pore-like regions using distance-transform and watershed-based methods. Quantitative metrics including pore volume, surface area, solidity, extent, and sphericity were extracted to characterize local structural remodeling of the lung parenchyma. Detailed segmentation and analysis procedures are described in the Supplementary Methods.

### Guided sectioning and histology

Three-dimensional phase-contrast micro-CT datasets were used to guide sectioning toward structurally altered regions within the FFPE lung tissue. Adjacent 5 µm sections were prepared for histology and AFM measurements. H&E-stained sections were digitized and scored for consolidation and lung injury using blinded semi-quantitative assessment. Detailed sectioning, imaging, and scoring procedures are provided in the Supplementary Methods.

### Atomic force microscopy

Atomic force microscopy (AFM) force spectroscopy was performed on deparaffinized FFPE lung sections to quantify local tissue stiffness in alveolar parenchyma and consolidated regions. Young’s modulus was estimated using Hertz-model fitting of force–distance curves. AFM measurements were spatially co-registered with phase-contrast micro-CT and histology to enable correlative structural and biomechanical analysis. Detailed sample preparation and acquisition parameters are provided in the Supplementary Methods.

### Spatial registration and correlation analysis

Spatial co-registration between phase-contrast micro-CT, histology, and AFM datasets was performed using elastic registration approaches adapted from D’Amico et al. [10]. Spatial relationships between fibrotic regions and enlarged pores were assessed using three-dimensional k-nearest neighbor analysis combined with bootstrap resampling. Detailed implementation and statistical procedures are described in the Supplementary Methods.

### Software and statistics

Statistical analysis was performed using Python-based scientific computing tools including Seaborn [11], SciPy [12], and scikit-learn [13]. Group-wise comparisons were performed using two-sided Mann–Whitney–Wilcoxon tests with Benjamini–Hochberg correction for multiple testing. Correlation analysis was performed using Spearman correlation. Adjusted p-values below 0.05 were considered statistically significant. Additional details are provided in the Supplementary Methods.

### Summary of the study design

**Fig. 1:**
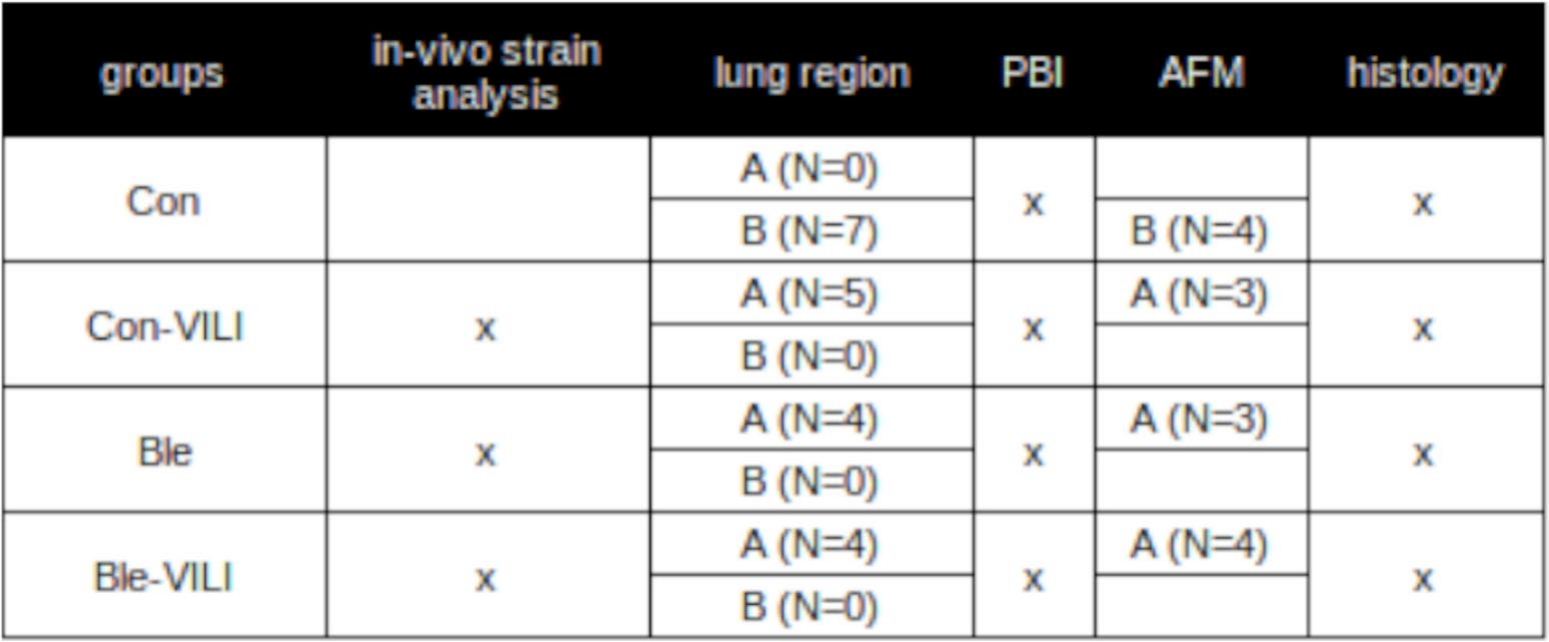
Overview of the study design. For ex-vivo analysis, the left lung lobe was divided into upper (A) and lower (B) regions prior to paraffin embedding. Con=healthy controls after protective ventilation; Con-VILI=healthy controls after injurious ventilation; Bleo=bleomycin-treated lungs after protective ventilation; Bleo-VILI=bleomycin-treated lungs after injurious ventilation. PBI=propagation-based imaging; AFM=atomic force microscopy

A schematic overview of the experimental workflow, imaging pipeline, and analysis strategy is shown in Fig. 1.

## Results

### Design of the data acquisition and analysis pipeline

**Fig. 2:**
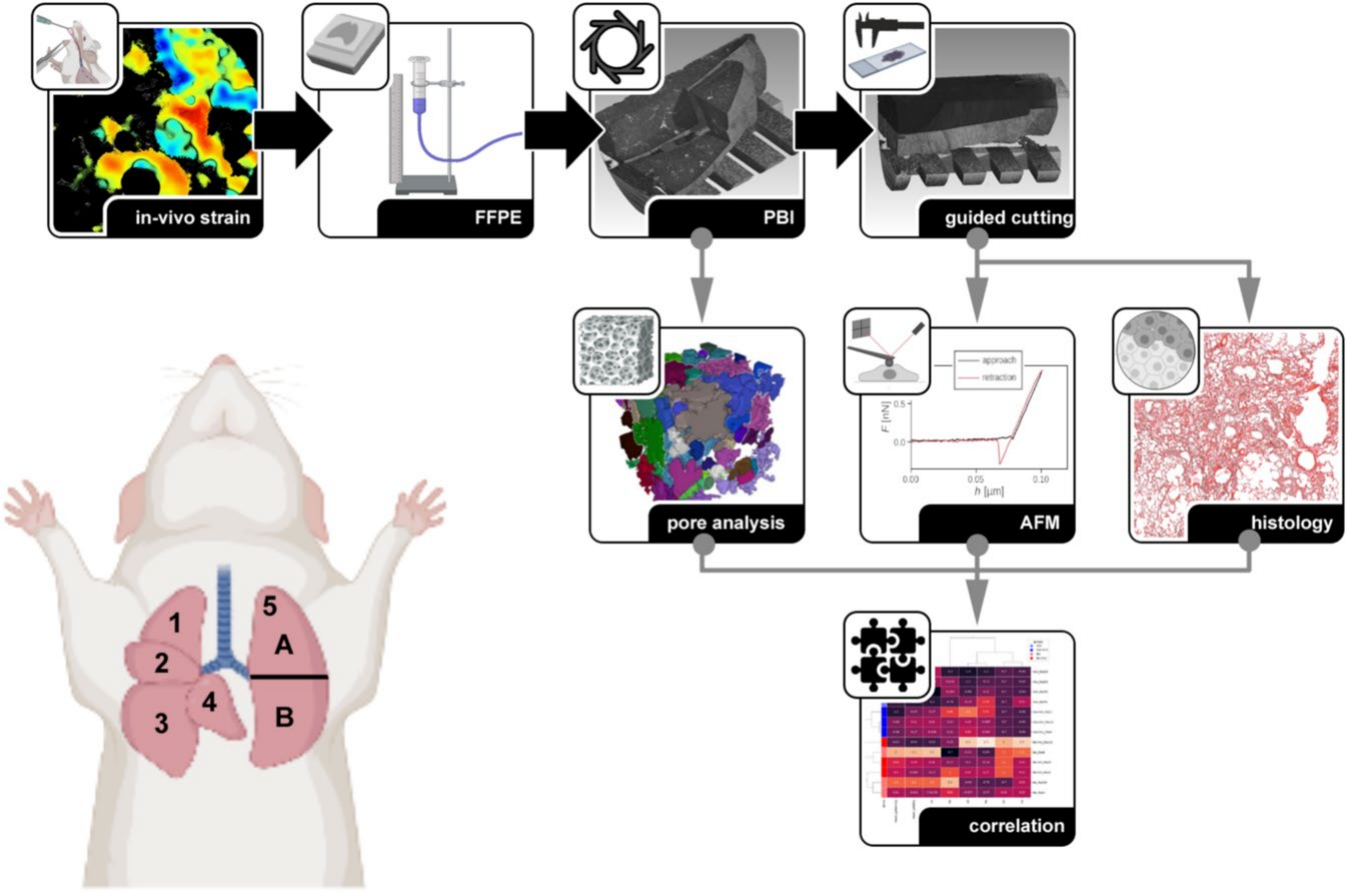
Experimental and data analysis workflow. Following in-vivo imaging and ventilation experiments, lungs were harvested, fixed, and embedded in paraffin. FFPE specimens were imaged using propagation-based phase-contrast micro-CT for three-dimensional structural analysis and guided sectioning. Corresponding tissue sections were analyzed using AFM and histology. Structural, biomechanical, and histological datasets were subsequently integrated for multiscale correlation analysis

To investigate the relationship between ventilator-induced lung injury (VILI) and bleomycin-induced lung remodeling, we established a multiscale correlative workflow integrating in-vivo respiratory mechanics and strain mapping with ex-vivo structural and biomechanical analysis (Fig. 2). Bleomycin-treated and control animals underwent protective or injurious mechanical ventilation protocols prior to tissue harvesting. Following fixation and paraffin embedding, FFPE lung specimens were imaged using synchrotron phase-contrast micro-CT to enable three-dimensional structural analysis and guided sectioning. Corresponding tissue sections were subsequently analyzed using histology and atomic force microscopy (AFM). The resulting datasets were spatially integrated to correlate airspace remodeling, local tissue stiffness, and histological injury patterns across scales.

### Results of the pore analysis and force-spectroscopy–based AFM

**Fig. 3:**
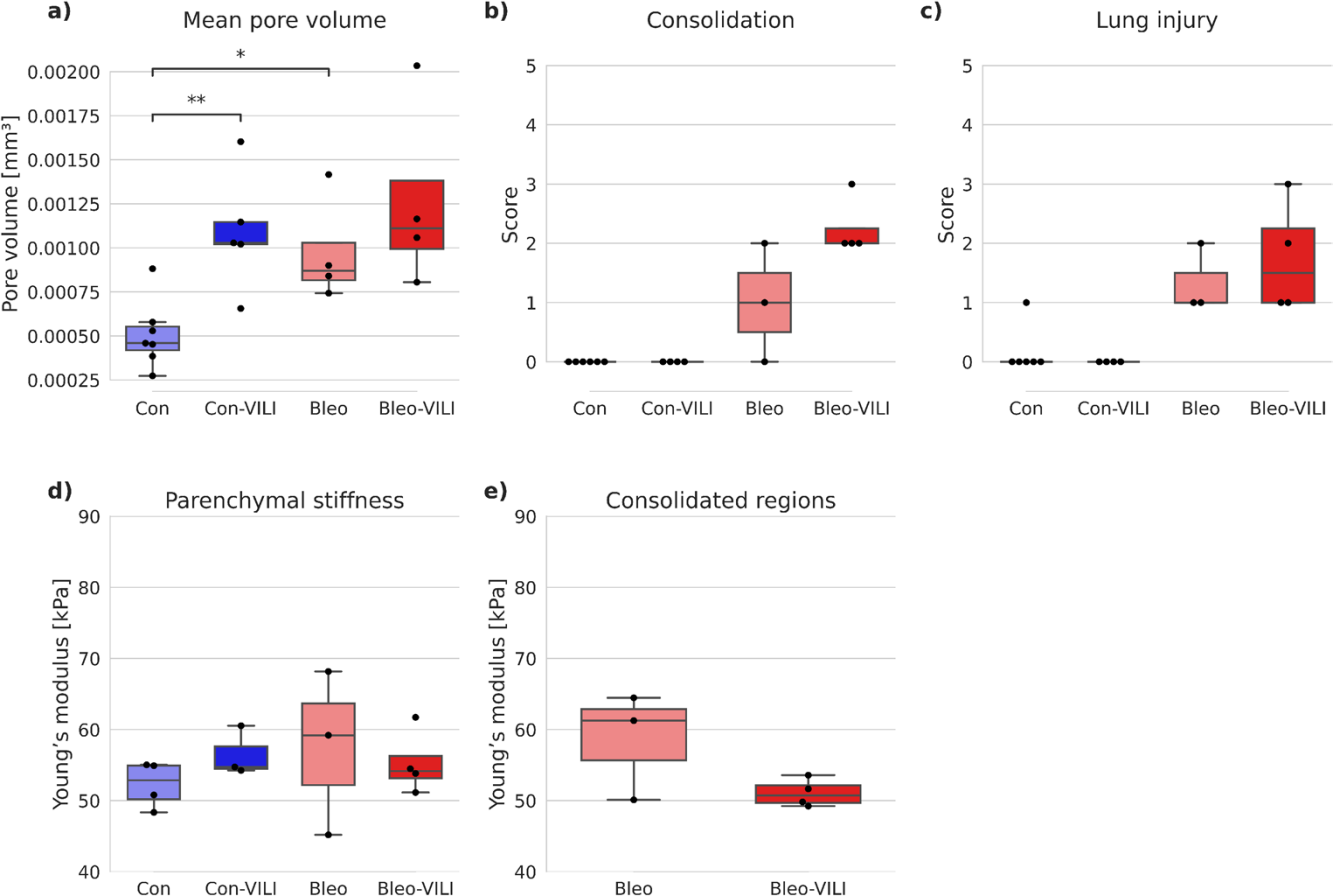
Quantitative results. **a** A significant increase in average pore volume was observed between healthy controls without VILI (Con) and those with VILI (Con-VILI), as well as between controls (Con) and bleomycin-induced lung injury (Bleo). **b** Histological scoring demonstrated increased consolidation in bleomycin-treated lungs, with higher scores observed in the Bleo-VILI group compared to the Bleo group. **c** Lung injury scores were elevated in bleomycin-treated groups compared to controls, whereas only minor differences were observed between Con and Con-VILI lungs. **d** AFM measurements of alveolar regions revealed no significant differences in parenchymal stiffness between groups. **e** In consolidated regions, a trend toward reduced stiffness was observed in Bleo-VILI lungs compared to Bleo lungs

Automated three-dimensional pore analysis and AFM force spectroscopy revealed marked structural and biomechanical differences between the experimental groups. All measurements were averaged for each individual rat and are presented in Fig. 3. A significant increase in average pore volume was observed both between healthy controls without VILI (Con) and healthy controls after injurious ventilation (Con-VILI), as well as between healthy controls and bleomycin-treated lungs (Bleo) (Fig. 3a).

Histological scoring demonstrated increased consolidation in the bleomycin-treated groups, with higher consolidation scores observed in Bleo-VILI compared to Bleo specimens (Fig. 3b). In contrast, no consolidation was observed in the control groups. Lung injury scores were elevated in both bleomycin-treated groups compared to controls, while only minor differences were observed between Con and Con-VILI lungs (Fig. 3c). These findings differed from the pore analysis results, which detected significant airspace enlargement in the Con-VILI group, highlighting differences between two-dimensional histological assessment and three-dimensional structural quantification.

No significant differences in alveolar wall stiffness were observed between the experimental groups (Fig. 3d). In consolidated regions, a trend toward reduced stiffness in the presence of VILI was observed in Bleo-VILI specimens compared to Bleo specimens (Fig. 3e).

**Fig. 4:**
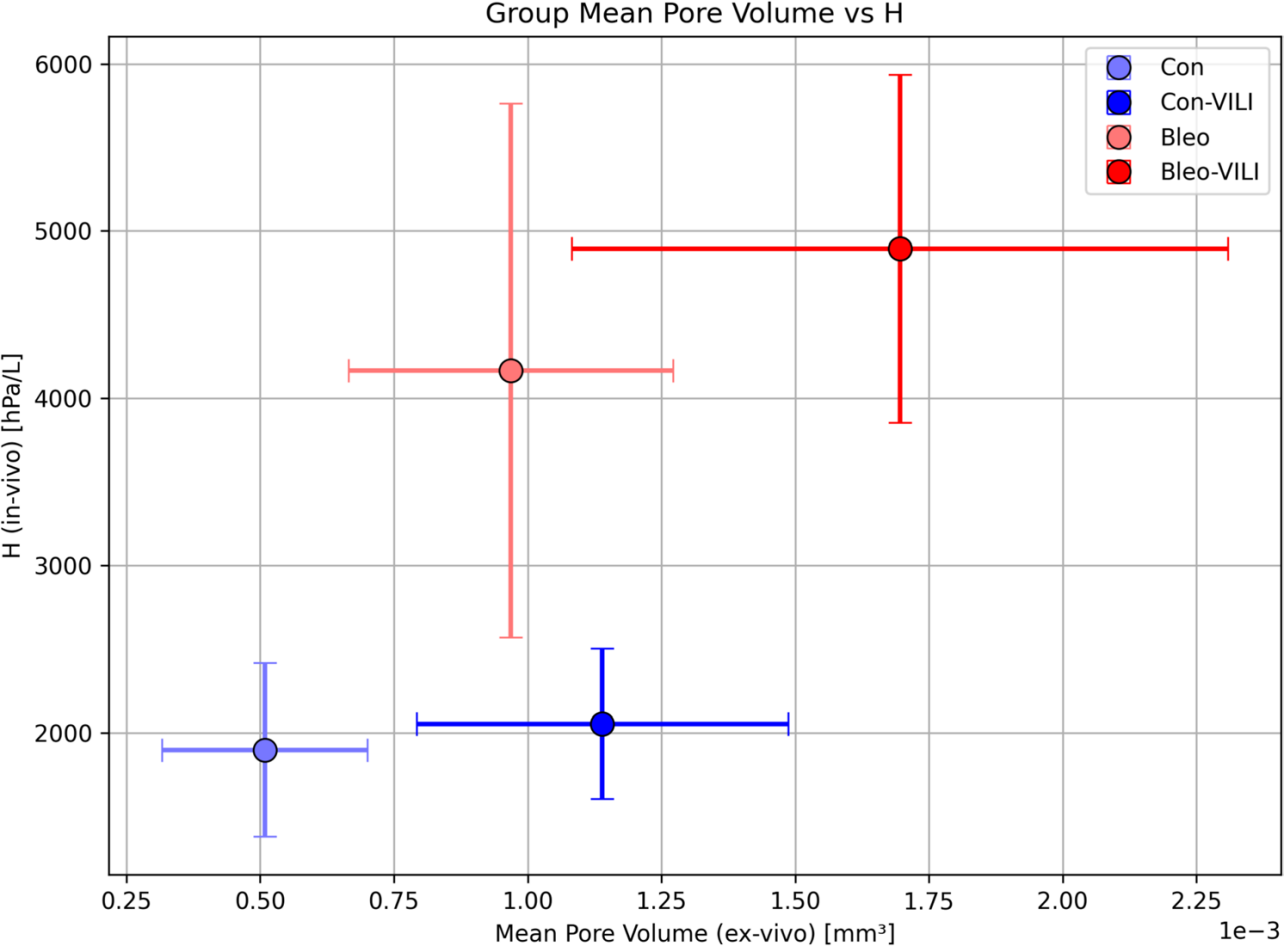
Relation between global lung elastance and pore volume. Global respiratory elastance (H) [7] and pore volume both increased following injurious mechanical ventilation in healthy control lungs (Con-VILI) and in bleomycin-treated lungs (Bleo, Bleo-VILI)

Comparison of in-vivo respiratory mechanics with ex-vivo pore analysis demonstrated that global tissue elastance (H) increased together with mean pore volume following injurious mechanical ventilation and after bleomycin-induced injury (Fig. 4).

**Fig. 5:**
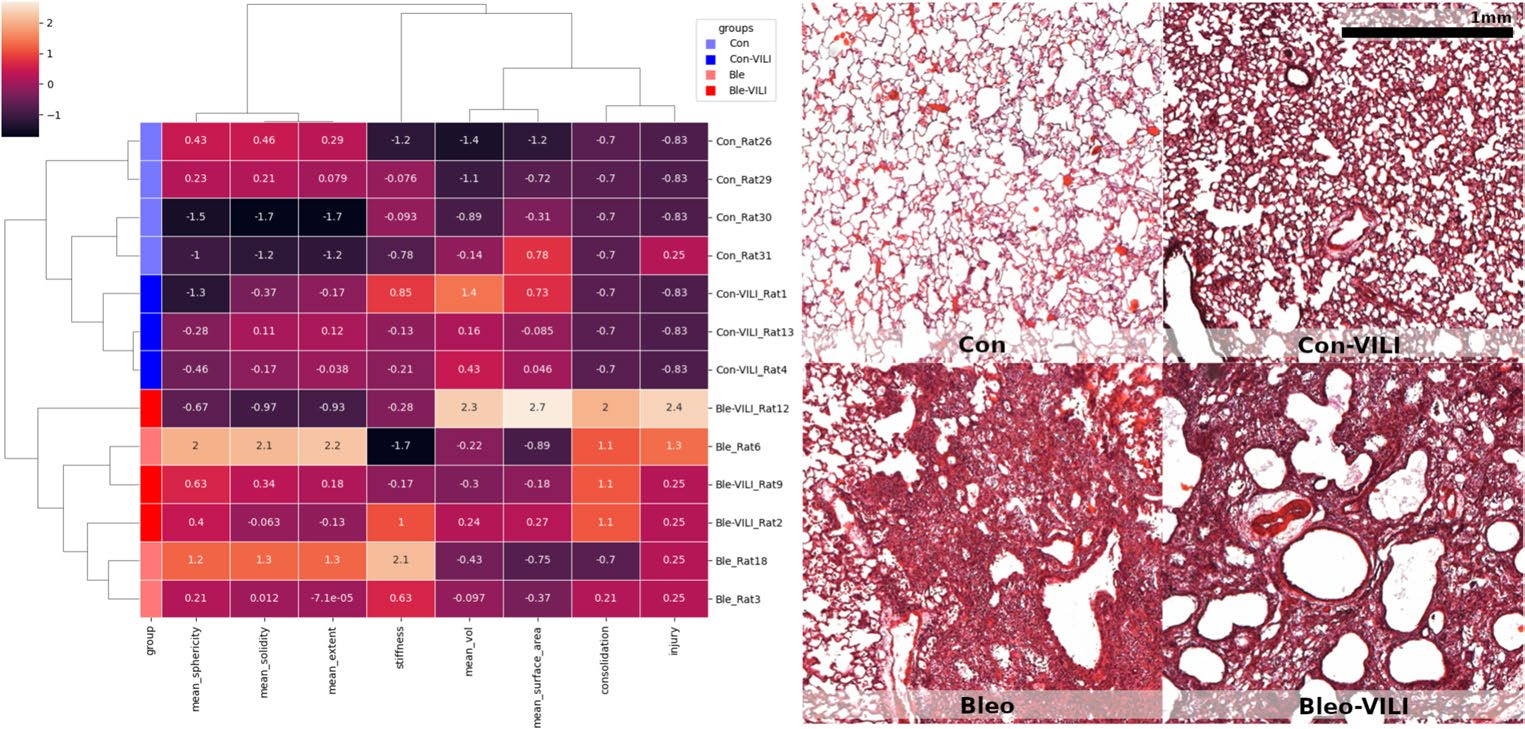
Cluster analysis. Hierarchical clustering based on pore-analysis metrics, AFM measurements, and histological scores separated healthy control lungs (Con, Con-VILI) from specimens with early fibrotic remodeling (Bleo, Bleo-VILI). Separation between protective and injurious ventilation conditions was more pronounced in control lungs than in bleomycin-treated lungs. Histological variables, particularly consolidation and lung injury scores, contributed strongly to the clustering structure and reflected marked differences in tissue remodeling across groups

To assess whether the experimental groups exhibited distinct combinations of structural, biomechanical, and histological features, hierarchical clustering was performed using z-score normalization, cosine similarity, and average linkage criteria (Fig. 5). The analysis clearly separated non-fibrotic lungs from specimens with early fibrotic remodeling. Separation between protective and injurious ventilation conditions was more pronounced in control lungs than in bleomycin-treated lungs, consistent with the pore analysis findings. Histological scoring variables contributed strongly to the clustering structure, reflecting marked differences in consolidation and tissue injury across the experimental groups. In contrast, VILI-associated alterations in the Con-VILI group were detected more prominently by three-dimensional pore analysis than by conventional histological scoring.

### Correlation and spatial integration of the results

**Fig. 6:**
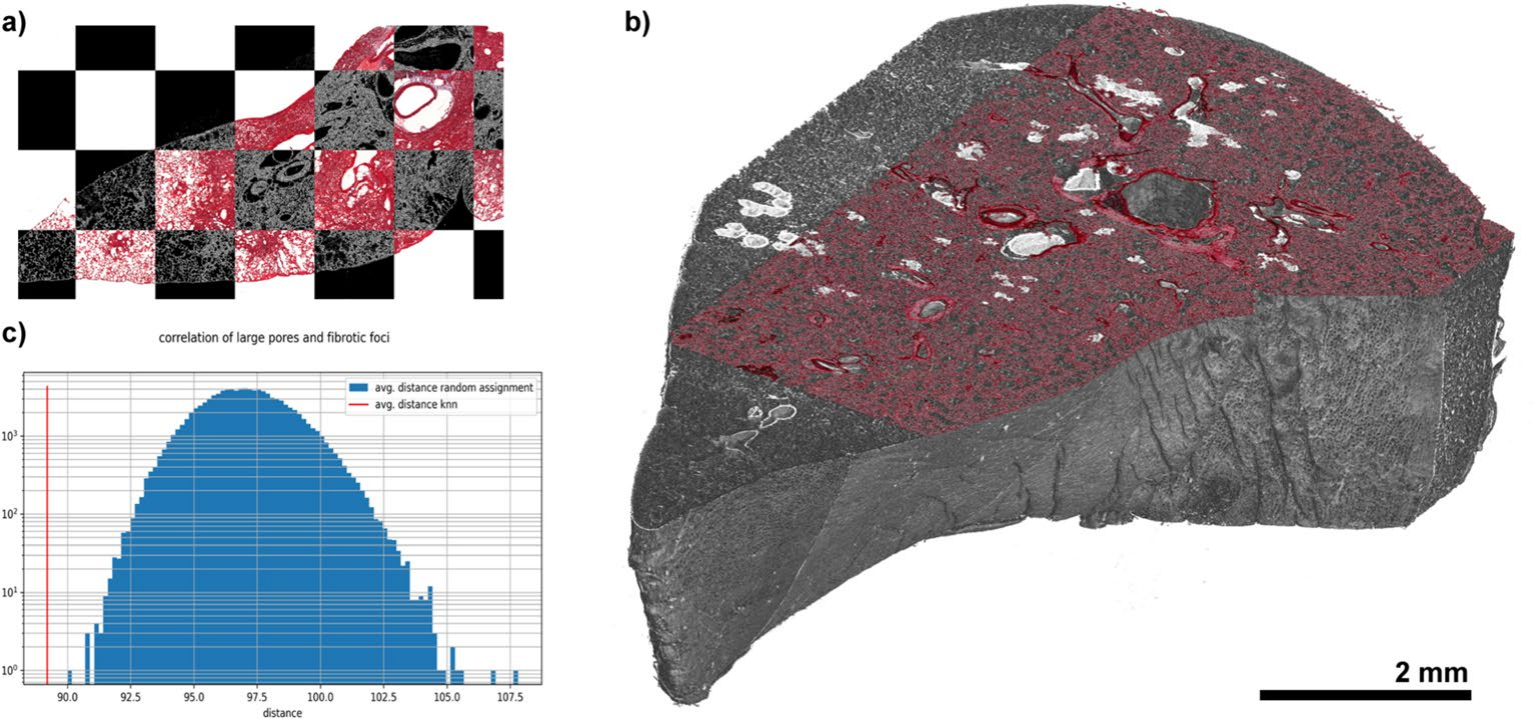
Spatial integration and correlation of Bleo-VILI lungs. **a** Checkerboard visualization of the elastic registration between a virtual cutting plane extracted from a phase-contrast micro-CT dataset and the corresponding H&E-stained histological section, demonstrating accurate spatial alignment. **b** Three-dimensional rendering of the phase-contrast micro-CT dataset with integrated histological information after spatial registration. **c** k-nearest neighbor (kNN) analysis demonstrated significantly smaller distances between fibrotic regions and enlarged pores than expected from bootstrap-resampled datasets, indicating spatial co-localization between fibrotic remodeling and ventilation-associated structural damage

Spatial co-registration enabled direct comparison of three-dimensional phase-contrast micro-CT datasets with corresponding histological and AFM measurements (Fig. 6a,b). Elastic registration demonstrated high alignment accuracy between reconstructed virtual cutting planes and histological sections, allowing structural alterations identified in the micro-CT datasets to be compared with corresponding histological features.

To investigate whether fibrotic remodeling spatially correlated with ventilation-induced structural damage, three-dimensional k-nearest neighbor analysis was performed between fibrotic regions and enlarged pores. The analysis demonstrated significantly smaller nearest-neighbor distances than expected from bootstrap-resampled datasets (Fig. 6c), indicating spatial co-localization between fibrotic regions and enlarged airspaces.

## Discussion

In this study, we established a multiscale correlative workflow combining label-free phase-contrast micro-CT, atomic force microscopy (AFM), and histology for quantitative assessment of lung structure and mechanics in FFPE rat lungs. Using this approach, we investigated how ventilator-induced lung injury (VILI) interacts with bleomycin-induced inflammation and early extracellular matrix (ECM) remodeling. Automated pore analysis, image registration, and cluster analysis enabled objective quantification of structural and mechanical alterations across spatial scales.

Our results demonstrate a significant increase in average pore volume, indicative of microscale airspace enlargement, following injurious ventilation in healthy lungs as well as after bleomycin-induced injury with early matrix remodeling. Notably, airspace enlargement was most pronounced in bleomycin-injured lungs exposed to injurious ventilation.

Post-mortem histology from the pre-protective ventilation era revealed bronchiolar dilatation and alveolar overdistension in ARDS patients ventilated with high tidal volumes and pressures, resembling emphysematous changes [14]. Similar findings have been reproduced experimentally in mechanically ventilated pigs with induced pneumonia [15, 16]. Airspace enlargement has also been reported in chronic inflammatory models including endotoxemia [17], oxygen toxicity [18], and starvation [19]. However, whether mechanical stress alone can induce persistent structural enlargement remains controversial [14].

Our data further show that early bleomycin-induced lung injury (day 7) is associated with airspace enlargement. This may reflect inflammatory elastin degradation, as neutrophil elastase released during type I inflammation can promote septal destruction [20, 21]. Alternatively, matrix deposition and stiffening during early remodeling may increase radial traction forces on alveolar walls, promoting mechanical dilation analogous to retraction bronchiectasis in fibrotic lung disease [22]. These mechanisms are not mutually exclusive and may coexist during early remodeling.

Importantly, our findings suggest that high-strain mechanical ventilation induces microscale airspace enlargement even in initially healthy lungs. While extreme ventilatory pressures can cause overt structural failure such as interstitial emphysema and pneumothorax [23], more subtle matrix-mediated mechanisms are likely operative at lower strain levels. The ECM transmits mechanical forces to resident cells and activates mechanotransductive pathways that amplify inflammatory responses [24]. Qualitative histology revealed inflammatory infiltration and matrix thickening in ventilated lungs, consistent with this interpretation. Together with the concomitant increase in global respiratory elastance, these findings suggest that altered matrix mechanics and inflammation, rather than acute septal rupture, contribute to the observed microscale enlargement. Notably, these structural changes are not detectable clinically or by conventional CT imaging.

A counter-intuitive finding of our AFM measurements was the trend toward reduced local stiffness in consolidated regions of Bleo-VILI lungs despite the overall increase in global respiratory elastance. While established fibrosis is generally associated with tissue stiffening [25], the early bleomycin phase studied here is dominated by inflammatory and oedematous remodeling rather than mature collagen deposition. Recent AFM studies have demonstrated a non-monotonic stiffness trajectory with an initial softening phase during early bleomycin injury [26]. Several non-exclusive mechanisms may explain why injurious ventilation accentuates this transient softening. Consolidated regions contain protein-rich edema and inflammatory infiltrates that are mechanically softer than organized ECM; alveolar collapse removes prestress from the elastin-collagen network; and high-strain ventilation can trigger proteolytic remodeling through neutrophil elastase and MMP activity [27–29]. The observed regional softening is therefore best interpreted as a composite effect of edema, collapse, cellular infiltration, and protease-mediated matrix disruption superimposed on an immature fibrotic matrix.

The workflow presented here is particularly suited to studying how ECM remodeling modifies susceptibility to ventilation-induced injury. This question is clinically relevant in both pulmonary fibrosis and fibroproliferative ARDS, where heterogeneous tissue mechanics are thought to generate regional stress concentrations during mechanical ventilation [30, 31]. In our Bleo-VILI cohort, regions with early fibrotic changes co-localized with the most pronounced ventilation-associated microstructural damage, providing a mechanistic framework for investigating how local stiffness heterogeneity influences injury propagation. Likewise, longitudinal application of this workflow after acute ventilator injury could help identify structural and biomechanical signatures associated with progression toward fibroproliferative remodeling following ARDS [32, 33]. Because our study is cross-sectional, however, it cannot determine whether the observed changes predict subsequent fibrosis progression.

Our results align with recent nanoscale studies demonstrating strain- and bleomycin-dependent collagen remodeling in the same animals [7]. By linking global in-vivo respiratory mechanics to microscale airspace enlargement and local stiffness alterations, the present study provides a multiscale framework connecting organ-level dysfunction to ECM remodeling. In contrast to biochemical assays that quantify bulk matrix composition, the combined micro-CT–AFM–histology approach enables spatially resolved assessment of the structural and mechanical consequences of remodeling.

Phase-contrast micro-CT combined with guided sectioning enabled quantitative 3D pore analysis in FFPE tissue [10, 34], while AFM provided local measurements of alveolar wall stiffness [10]. The direct integration of volumetric imaging, biomechanical characterization, and histological validation within a single correlative workflow represents a key methodological strength. Importantly, this approach mitigates limitations of conventional histology arising from sampling bias and the inherently two-dimensional representation of heterogeneous lung injury [34, 35].

The use of automated pore analysis proved particularly sensitive for detecting ventilation-induced structural damage. Significant alterations were identified in healthy ventilated lungs even when classical histology showed minimal differences, highlighting the advantages of objective three-dimensional quantification. Nevertheless, pore metrics remain abstractions of the hierarchical airway network and may be affected by segmentation and FFPE-related artifacts [36].

Several limitations warrant consideration. First, FFPE tissue alters mechanical properties due to formalin-induced cross-linking [37], and AFM measurements on FFPE are generally less sensitive than measurements on fresh tissue [38]. Second, rat lungs were too large to embed intact in standard paraffin blocks, requiring dissection into upper and lower lobes. Consequently, PBI scans captured only parts of the lung, preventing normalization of pore metrics to total lung volume. Third, air inclusions in FFPE specimens produced artifacts in PBI scans, necessitating selective ROI analysis [39]. Only the Bleo-VILI specimens originated from the identical animals subjected to ex-vivo analysis in the present study, limiting direct in-vivo–ex-vivo correlations to this cohort. Furthermore, although short-term high-volume ventilation primarily induces mechanical injury, it also produces inflammatory changes including interstitial thickening, neutrophil infiltration, and cytokine release [40]. In contrast, early bleomycin injury generates a stronger inflammatory response with edema and fibroproliferative activity emerging around day 7 [41]. Because our study is cross-sectional, it cannot identify markers predictive of subsequent fibroproliferation; longitudinal studies will be required to address this question. Finally, the bleomycin model exhibits substantial spatial heterogeneity, complicating comparisons between groups [42].

Despite these limitations, our findings are consistent with previous in-vivo observations demonstrating reduced VILI susceptibility in lungs with early fibrotic changes [43]. Together with nanoscale evidence of collagen remodeling [7], the observed co-localization of regions with early fibrotic changes and enlarged pores supports the concept that ECM remodeling alters regional mechanical behavior and stress propagation. The presented multimodal workflow therefore provides a complementary approach for linking molecular remodeling processes to their structural and biomechanical consequences.

Conventional histopathological scoring remains an important reference standard in experimental VILI research but is limited by observer dependence, semiquantitative grading, and sampling bias [44]. Rather than replacing histology, the correlative micro-CT–AFM workflow may complement future pathology-based assessment by providing automated, volumetric, and spatially resolved measurements of structural remodeling and tissue mechanics that are inaccessible to conventional two-dimensional analysis.

## Conclusion

We implemented a multiscale correlative workflow combining label-free phase-contrast micro-CT, automated pore analysis, atomic force microscopy, and histology to characterize structural and mechanical alterations in lung injury. Both injurious mechanical ventilation and bleomycin-induced inflammatory remodeling promoted microscale airspace enlargement, with the strongest effects observed in the combined Bleo-VILI group. Spatial co-localization of regions with early fibrotic changes and ventilation-induced structural damage suggests that pre-existing ECM remodeling modifies regional stress distribution and injury propagation. By linking global respiratory mechanics to microscale architecture and previously reported nanoscale collagen remodeling [7], this approach provides a multiscale framework for investigating interactions between extracellular matrix remodeling and ventilator-induced lung injury.

## Additional file

**Additional file 1.** Supplementary Material 1.

## Data Availability

The datasets used and analyzed during the current study are available from the corresponding author on reasonable request.

## Abbreviations

2D: Two-dimensional
3D: Three-dimensional
4D: Four-dimensional
AFM: Atomic force microscopy
Ble: Bleomycin-induced lung injury rat model after protective mechanical ventilation
Ble-VILI: Bleomycin-induced lung injury rat model after injurious mechanical ventilation
Con: Healthy control rats after protective mechanical ventilation
Con-VILI: Healthy control rats after injurious mechanical ventilation
ESRF: European Synchrotron Radiation Facility
FFPE: Formalin-fixed, paraffin-embedded
FOT: Forced oscillation technique
G: Tissue damping
GGG: Gallium Gadolinium Garnet
H: Dynamic tissue elastance
H&E: Hematoxylin and eosin
kNN: k-nearest neighbor
micro-CT: Micro-computed tomography
MV: Mechanical ventilation
ns: Not significant
PBI: Propagation-based imaging
PEEP: Positive end-expiratory pressure
Pip: Peak inspiratory pressure
Raw: Airway resistance
ROI: Region of interest
ROIs: Regions of interest
STP: SYRMEP Tomo Project
SYRMEP: SYnchrotron Radiation for Medical Physics
VILI: Ventilator-induced lung injury
VT: Tidal volume
