# Supplementary material for: Extracellular matrix remodeling modifies structural responses to ventilator-induced lung injury: a multiscale correlative imaging study
Source: Respir Res. 2026 Jul 13;27:287. doi: 10.1186/s12931-026-03807-y (PMC13383386; doi:10.1186/s12931-026-03807-y)
Supplement: Supplementary file 1 — Additional file 1. Supplementary Material 1. [file 12931_2026_3807_MOESM1_ESM.pdf]

Supplementary Methods
Extracellular matrix remodeling modifies structural responses to ventilator-induced lung injury: a multiscale correlative imaging study

Supplementary Methods

S1 Experimental ventilation settings and animal preparation

The experiments were performed on 20 Sprague-Dawley rats with an average body weight of 399±26 g. Animals were divided into four experimental groups: healthy controls after protective mechanical ventilation (Con), healthy controls after injurious ventilation (Con-VILI), bleomycin-treated animals after protective ventilation (Bleo), and bleomycin-treated animals after injurious ventilation (Bleo-VILI).

Bleomycin-induced lung injury was established by intratracheal instillation of 1000 iU bleomycin (Sigma-Aldrich, St. Louis, MO, USA) dissolved in 200 µl saline. Control animals received saline only. Animals were studied seven days after instillation.

For the *in-vivo* experiments, animals were anesthetized, tracheostomized, and mechanically ventilated under controlled conditions as described previously by Deyhle et al. [1]. Injurious mechanical ventilation was induced using pressure-controlled ventilation with peak inspiratory pressures of approximately 41±2 cm H_2O, zero positive end-expiratory pressure (PEEP), and a respiratory rate of 30 breaths per minute for 20 minutes.

At the end of the imaging experiments, animals were euthanized by intraperitoneal injection of pentobarbital sodium (Dolethal, 200 mg/kg, Vetoquinol, Lure, France). The heart and lungs were excised en bloc. The lungs were inflated with 4% paraformaldehyde at a constant pressure of 20 cm H_2O and fixed overnight. Subsequently, the tissue was dehydrated using a graded ethanol series and embedded in paraffin.

For further analysis, the left lung lobe was divided into an upper and lower region prior to paraffin embedding.

S2 Synchrotron phase-contrast microCT acquisition

All formalin-fixed paraffin-embedded (FFPE) lung specimens were scanned at the SYRMEP beamline [2] using propagation-based synchrotron phase-contrast microCT.

Imaging was performed using a white/pink beam configuration with a sample-to-detector propagation distance of 150 mm. A 0.5 mm silica filter was used, resulting in a mean X-ray energy of 16.7 keV. Off-center scans covering 360° were acquired using 3600 projections with an exposure time of 50 ms per projection.

Projection images were recorded using a water-cooled Orca Flash 4.0 sCMOS detector coupled to a 17 µm Gallium Gadolinium Garnet scintillator. The effective isotropic voxel size after reconstruction was 2 µm.

To capture the full FFPE specimen volume, 2–4 vertically overlapping scans were acquired for each paraffin block.

S3 Reconstruction and phase retrieval

Tomographic reconstruction was performed using SYRMEP Tomo Project (STP) software [3]. Prior to reconstruction, phase retrieval was applied using the single-distance transport of intensity equation homogeneous object approximation (TIE-Hom) method [4] with a δ/β ratio of 100.

Filtered back-projection reconstruction was subsequently performed to generate three-dimensional datasets. Vertically overlapping scans were stitched using a custom Python-based workflow to obtain complete reconstructed specimen volumes.

S4 ROI extraction and pore segmentation

Automated pore analysis was performed using PoreSpy [5]. Cubic regions of interest (ROIs) of 300 × 300 × 300 voxels were extracted from the reconstructed datasets using an overlap of 100 voxels between adjacent ROIs.

ROIs intersecting the reconstructed field-of-view boundary or containing visible air artifacts were excluded from further analysis.

Segmentation of airspaces was performed using global thresholding with an empirically determined threshold value of 55. Morphological hole filling and binary closing operations were subsequently applied to reduce segmentation artifacts.

Because the airway network is fully connected, a Euclidean distance transform was computed on the segmented airspaces. Watershed-based separation was then performed following thresholding of the distance map at 10 voxels to divide the connected airway space into isolated pore-like regions.

For each segmented pore, the following quantitative parameters were calculated:

$$\text{Pore volume} = \text{number of segmented voxels} \times \text{voxel volume} \tag{1}$$

$$\text{Solidity} = \frac{\text{pore volume}}{\text{convex hull volume}} \tag{2}$$

$$\text{Extent} = \frac{\text{pore volume}}{\text{bounding box volume}} \tag{3}$$

Surface area was estimated using triangulated surface approximations generated from the segmented pore boundaries. A 2D illustration showing the geometric expressions of these features is provided in Suppl. Fig. 1.”**

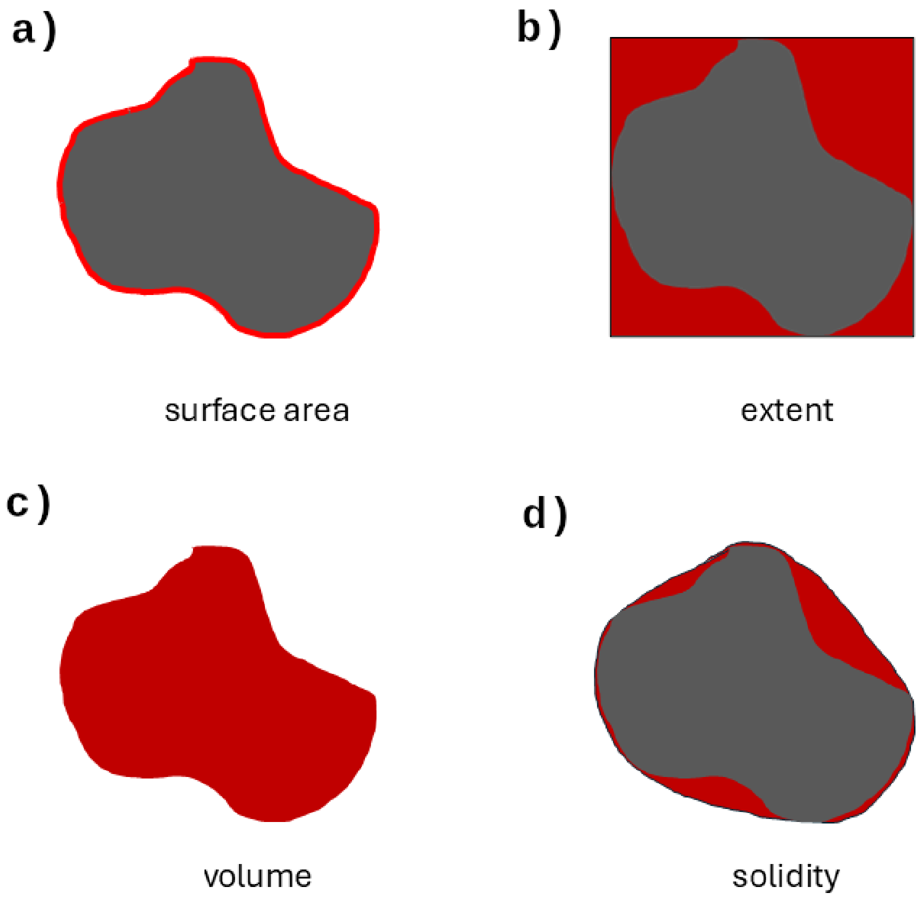

Suppl. Fig. 1 Geometric representations of (a) the surface area, (b) the extent, (c) the volume, and (d) the solidity of a segmented pore.

S5 Guided sectioning and AFM sample preparation

Three-dimensional phase-contrast microCT datasets were imported into VGSTUDIO MAX (Volume Graphics, Germany) for guided sectioning and localization of regions of interest.

Datasets were aligned to the paraffin block surface to estimate the cutting depth required for targeting fibrotic and structurally altered regions. Because paraffin blocks were sectioned under cooled conditions whereas scans were acquired at room temperature, an empirically determined shrinkage correction factor of 20% was applied.

Adjacent 5 µm tissue sections were prepared. One section was stained using hematoxylin and eosin (H&E), while an adjacent unstained section was used for AFM measurements.

For AFM measurements, sections were deparaffinized and rehydrated according to the following protocol:

- 1 hour at 65°C
- 15 minutes in xylol
- rinse in fresh xylol
- 10 minutes in 99% ethanol
- 10 minutes in 96% ethanol
- 5 minutes in 50% ethanol
- transfer to distilled water

S6 AFM acquisition and analysis

AFM measurements were performed using a Nanowizard 4 system (JPK, Bruker Nano GmbH) mounted on an Olympus IX83 inverted microscope.

Force spectroscopy measurements were acquired in water using pre-calibrated SiO_2 cantilevers with a spring constant of 0.062 N/m and a spherical bead radius of 2.5 µm.

Measurements were acquired using a setpoint force of 10 nN and a z-axis approach speed of 2 µm/s.

Young's modulus values were extracted using JPK Data Processing software (version 7.0.165). Baseline subtraction and contact point determination were applied prior to fitting. Tissue elasticity was estimated using the Hertz contact model [6], assuming homogeneous elastic material behavior and a non-deformable spherical indenter.

A custom Python workflow was used to spatially overlay AFM measurements with microscopy images and corresponding phase-contrast microCT datasets.

S7 Histological scoring workflow

H&E-stained sections were digitized using an Axioskop microscope (Zeiss) equipped with a Micropublisher 5.0 camera (QImaging Surrey). Whole-slide mosaic scans were acquired at 5× magnification.

Images were subdivided into tiles of 1024 × 1204 pixels. Tiles containing less than 25% tissue coverage were excluded from analysis.

The remaining image tiles were randomized and independently scored for:

- extent of consolidation
- degree of lung injury

Scoring was performed using a four-point ordinal scale ranging from:

- 0 = absent
- 1 = mild
- 2 = moderate
- 3 = severe

Readers were blinded to experimental group assignment and ventilation condition. Median scores per specimen were used for statistical analysis.

S8 k-nearest neighbor analysis and bootstrap statistics

To investigate spatial relationships between fibrotic regions and enlarged pores, a three-dimensional k-nearest neighbor (kNN) analysis was performed.

Fibrotic regions were identified using thresholded distance-transform-based segmentation of thickened tissue regions in the reconstructed phase-contrast microCT datasets. Centroids of these regions were extracted to generate the first point cloud.

The second point cloud consisted of centroids of segmented pores with volumes larger than 0.00064 mm^3.

For each point in the fibrotic-region point cloud, distances to the 10 nearest neighboring enlarged pores were calculated using scikit-learn (version 1.6.0) [7]. Mean nearest-neighbor distances were recorded.

Statistical significance was assessed using bootstrap resampling with replacement. A total of 100,000 bootstrap iterations were performed while maintaining the original point-cloud cardinalities.

The p-value was calculated as:

$$p = \frac{1}{N} \sum_{i=1}^{N} \mathbb{I}\left(\bar{d}_i^* \leq \bar{d}_{\text{obs}}\right) \tag{4}$$

where:
$\bar{d}_{\text{obs}}$ = observed mean kNN distance
$\bar{d}_i^*$ = bootstrap sample mean distance
$\mathbb{I}$ = indicator function

S9 Statistical analysis

Statistical analysis was performed using Seaborn (version 0.13.2) [8] together with statannotations (version 0.7.1) [9].

Group-wise comparisons were performed using two-sided Mann–Whitney–Wilcoxon tests with Benjamini–Hochberg correction for multiple testing.

A p-value below 0.05 was considered statistically significant.

Spearman correlation analysis was performed using SciPy (version 1.16.3) [10].

Hierarchical clustering was performed using z-score normalization, cosine similarity, and average linkage criteria.

References

[1] Deyhle Jr RT, et al. Nanoscale structural alteration of lung collagen in response to strain and bleomycin injury. Scientific Reports. 2025;15(1):21178.

[2] Longo E, Contillo A, D'Amico L, Prašek M, Saccomano G, Sodini N, et al. SYRMEP beamline: state of the art, upgrades and future prospects. The European Physical Journal Plus. 2024;139(10):880.

[3] Brun F, Pacilè S, Accardo A, Kourousias G, Dreossi D, Mancini L, et al. Enhanced and flexible software tools for X-ray computed tomography at the Italian

synchrotron radiation facility Elettra. Fundamenta Informaticae. 2015;141(2-3):233–243.

[4] Paganin D, Mayo SC, Gureyev T, Miller PR, Wilkins S. Simultaneous phase and amplitude extraction from a single defocused image of a homogeneous object. Journal of Microscopy. 2002;206(1):33–40. https://doi.org/10.1046/j.1365-2818.2002.01010.x.

[5] Gostick JT, et al. PoreSpy: A python toolkit. Journal of Open Source Software. 2019;4(37):1296.

[6] Kontomaris SV, et al. Hertz Model Limitations and Corrections for AFM Contact Mechanics. Measurement. 2020;155:107552.

[7] Pedregosa F, et al. Scikit-learn. JMLR. 2011;12:2825–2830.

[8] Waskom ML. seaborn: statistical data visualization. Journal of Open Source Software. 2021;6(60):3021. https://doi.org/10.21105/joss.03021.

[9] Charlier F, et al.: Statannotations.

[10] Virtanen P, Gommers R, Oliphant TE, Haberland M, Reddy T, Cournapeau D, et al. SciPy 1.0: Fundamental Algorithms for Scientific Computing in Python. Nature Methods. 2020;17:261–272. https://doi.org/10.1038/s41592-019-0686-2.
